# The Efficacy and Safety of Endoscopic Balloon Dilatation in the Treatment of Functional Post-Sleeve-Gastrectomy Stenosis

**DOI:** 10.3390/medicina60050833

**Published:** 2024-05-19

**Authors:** Mohamed A. Elsebaey, Mohamed Elsayed Enaba, Heba Elashry, Waleed Elrefaey, Rasha Youssef Hagag, Neveen A. Shalaby, Mohamed Sabry Aboelnasr, Mohamed Elsayed Sarhan, Omneya Mohamed Darrag, Assem Mohamed Elsokkary, Mohamed Abd Allah Alabd, Ahmed Mohamed El Nakib, Abdulrashid Onimisi Abdulrahim, Yousry Esam-Eldin Abo-Amer, Mohammad Shaaban Mahfouz, Amina Mahmoud Fouad, Raghda Samir Abd El latif, Khaled Asem Allam, Amro Abdelaziz Mohammed Ismail

**Affiliations:** 1 Internal Medicine Department, Faculty of Medicine, Tanta University, Tanta 31511, Egypt; mohamedelsebaey79@gmail.com (M.A.E.); m.enaba@hotmail.com (M.E.E.); dr.waleedelrefaey@gmail.com (W.E.); rasha.hagag@med.tanta.edu.eg (R.Y.H.); mohamed.aboelnasr@med.tanta.edu.eg (M.S.A.); aorta2025@yahoo.com (M.E.S.);; 2 Tropical Medicine Department, Faculty of Medicine, Tanta University, Tanta 31511, Egypt; 3 Internal Medicine Department, Mansoura New General Hospital, Mansoura 34008, Egypt; assemmelsokkary@gmail.com; 4 Gastroenterology, Hepatology and Infectious Diseases Department, Red Crescent Hospital, Tanta 66232, Egypt; 5 Tropical Medicine Department, Faculty of Medicine, Mansoura University, Mansoura 35516, Egypt; 6 Gastroenterology/Internal Medicine Department, Federal Medical Centre, Keffi 961101, Nasarawa State, Nigeria; 7 Hepatology, Gastroenterology and Infectious Diseases Department, Mahala Hepatology Teaching Hospital, El-Mahalla el-Kubra 31951, Egypt; 8 Hepatology, Gastroenterology and Infectious Diseases Department, Ahmed Maher Teaching Hospital, Cairo 11638, Egypt; mashhap8@gmail.com; 9 Clinical Pathology Department, National Hepatology and Tropical Medicine Research Institute, Cairo 42600, Egypt; 10 General Surgery Department, Ahmed Maher Teaching Hospital, Cairo 11638, Egypt; 11 Internal Medicine Department, Faculty of Medicine, Cairo University, Giza 12345, Egypt

**Keywords:** functional gastric stenosis, sleeve gastrectomy, balloon dilatation, clinical response

## Abstract

*Background and Objectives*: Functional gastric stenosis, a consequence of sleeve gastrectomy, is defined as a rotation of the gastric tube along its longitudinal axis. It is brought on by gastric twisting without the anatomical constriction of the gastric lumen. During endoscopic examination, the staple line is deviated with a clockwise rotation, and the stenosis requires additional endoscopic manipulations for its transposition. Upper gastrointestinal series show the gastric twist with an upstream dilatation of the gastric tube in some patients. Data on its management have remained scarce. The objective was to assess the efficacy and safety of endoscopic balloon dilatation in the management of functional post-sleeve gastrectomy stenosis. *Patients and Methods*: Twenty-two patients with functional post-primary-sleeve-gastrectomy stenosis who had an endoscopic balloon dilatation between 2017 and 2023 were included in this retrospective study. Patients with alternative treatment plans and those undergoing endoscopic dilatation for other forms of gastric stenosis were excluded. The clinical outcomes were used to evaluate the efficacy and safety of balloon dilatation in the management of functional gastric stenosis. *Results*: A total of 45 dilatations were performed with a 30 mm balloon in 22 patients (100%), a 35 mm balloon in 18 patients (81.82%), and a 40 mm balloon in 5 patients (22.73%). The patients’ clinical responses after the first balloon dilatation were a complete clinical response (4 patients, 18.18%), a partial clinical response (12 patients, 54.55%), and a non-response (6 patients, 27.27%). Nineteen patients (86.36%) had achieved clinical success at six months. Three patients (13.64%) who remained symptomatic even after achieving the maximal balloon dilation of 40 mm were considered failure of endoscopic dilatation, and they were referred for surgical intervention. No significant adverse events were found during or following the balloon dilatation. *Conclusions*: Endoscopic balloon dilatation is an effective and safe minimally invasive procedure in the management of functional post-sleeve-gastrectomy stenosis.

## 1. Introduction

Laparoscopic sleeve gastrectomy is one of the most common bariatric procedures performed globally, which is both technically simple and successful in treating morbid obesity [1,2,3]. Sleeve gastrectomy has complications, even though it seems to be a safe treatment [4]. Among these complications are hematoma and bleeding (1–5%), gastric stenosis (0.5–5%), and gastric leakage (1–4%) [5,6,7]. There are two types of post-sleeve-gastrectomy stenosis: organic and functional [8]. The anatomical stricture of the gastric tube is linked to organic stenosis [9]. In contrast, there is no actual constriction of the gastric lumen in functional stenosis, which is brought on by a twisting of the gastric tube along its longitudinal axis [10]. The pathogenesis of gastric twisting includes the disruption of the supporting ligaments of the stomach during sleeve gastrectomy, making the stomach more mobile and prone to twisting [11,12]. Unequal traction on the anterior and posterior gastric walls during the firing of the staples promotes the rotation of the stomach with a subsequent spiral pattern of stapling [13]. The third mechanism is the scarring and adhesion formation at the staple line, leading to the kinking of the gastric tube at the incisura angularis [14,15]. Patients’ quality of life is negatively impacted by post-sleeve-gastrectomy stenosis [16]. Patients present with obstructive gastric symptoms such as repeated vomiting, nausea, regurgitation, and post-prandial upper abdominal pain. It is worth noting that the prompt diagnosis and appropriate management of gastric stenosis minimize hazardous complications such as malnutrition, electrolyte disturbances, vitamin deficiencies, and repeated hospital admissions [17,18]. Currently, there is no clear consensus regarding the management of functional post-sleeve-gastrectomy stenosis, and the literature regarding the proper treatment of gastric twisting after sleeve gastrectomy is scarce [19]. Various therapeutic modalities, including conservative management, endoscopic, and surgical treatment, have been proposed as potential options [8,15,20,21]. Being a minimally invasive procedure, endoscopic balloon dilatation has emerged as a promising therapeutic option for gastric stenosis [20]. The aim of this study was to assess the efficacy and safety of endoscopic balloon dilatation in the management of symptomatic functional post-sleeve-gastrectomy stenosis. 

## 2. Patients and Methods

### 2.1. Study Design

**Figure 1:**
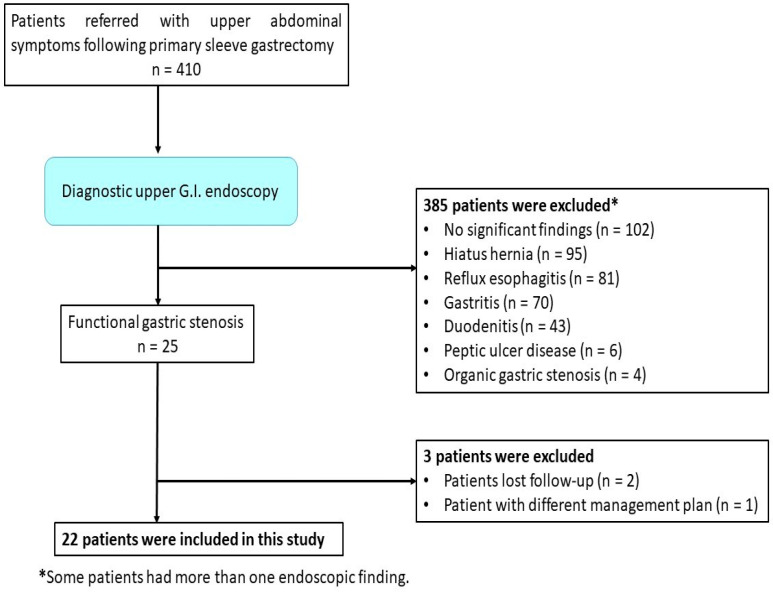
Flow chart of the selected patients.

This retrospective cohort study included 22 patients with functional post-sleeve-gastrectomy stenosis who underwent endoscopic balloon dilatation between January 2017 and May 2023. The study was conducted at the Internal Medicine Department of Tanta University Hospital, Tropical Medicine Department of Mansoura University Hospital, Mahala Hepatology Institute, and some private endoscopic referral centers in Tanta, Egypt. The study protocol was approved by the local research ethical committee of the faculty of medicine, Tanta University (approval code: 36264PR581/2/24). The research was conducted in compliance with the 1975 Helsinki Declaration’s ethical guidelines. A flow chart of the selected patients is shown in Figure 1.

### 2.2. Inclusion Criteria

The inclusion criteria were patients aged ≥18 years; with obstructive gastric symptoms following primary sleeve gastrectomy; and who were diagnosed to have functional gastric stenosis and underwent endoscopic balloon dilatation for this stenosis. The diagnosis of functional gastric stenosis was based on patients’ clinical symptoms, refractory to full-dose proton pump inhibitors, antiemetic drugs after 1–2 weeks [22], radiological imaging, and esophagogastroduodenoscopy (EGD) findings.

### 2.3. Exclusion Criteria

Patients who had endoscopic dilatation for other forms of gastric stenosis, including organic post-sleeve-gastrectomy stenosis, stenosis after bariatric surgeries other than sleeve gastrectomy, and post peptic strictures, were excluded from this study. Patients with alternative treatment plans, and those who did not complete a follow-up after the first balloon dilatation, were also ruled out.

### 2.4. Data Collection

The following data were collected from the patients’ medical files: age, sex, body mass index (BMI), comorbidities, date of sleeve gastrectomy, post-sleeve-gastrectomy presenting symptoms, hospital admission, results of laboratory investigations (complete blood count, liver function tests, renal function test, and serum electrolytes), diagnostic EGD findings, and details of the endoscopic balloon dilatation procedures. These details included the date of the first dilatation session, the achalasia balloon size used, the duration of each dilatation session, the total number of sessions, adverse events related to dilatation procedures, the short-term efficacy at 2 weeks following the first dilatation, and the presence of long-term efficacy (clinical success) at the 6-month follow-up period. 

### 2.5. Endoscopic Management

**Figure 2:**
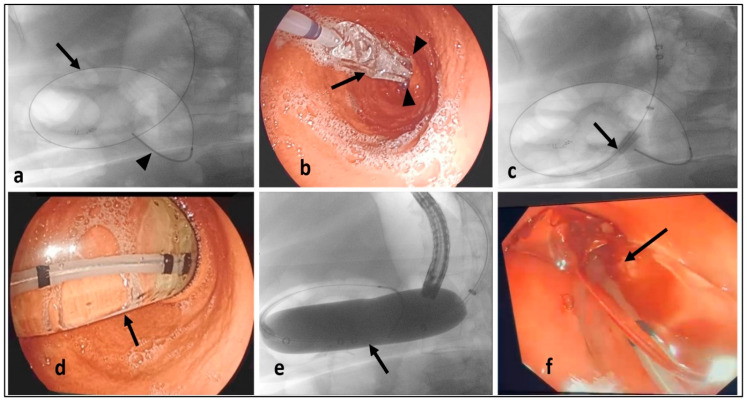
Steps of balloon dilatation of functional post-sleeve-gastrectomy stenosis. (**a**) Fluoroscopic visualization of the metallic guidewire (arrow) and its distal end (arrow head); (**b**) endoscopic image of passing the achalasia balloon (arrow) over the guidewire and its placement through the gastric stenosis at the incisura angularis (arrow heads); (**c**) fluoroscopic guidance while sliding the balloon (arrow) over the guidewire; (**d**) endoscopic image during the inflation of the balloon (arrow); (**e**) fluoroscopic confirmation of the inflated balloon position (arrow) across the gastric stenosis; and (**f**) endoscopic image during the deflation of the balloon with noticeable traces of blood (arrow).

All the patients underwent a diagnostic EGD and endoscopic balloon dilatation at the same session or within one week as an outpatient intervention without concomitant admission. However, some patients underwent the endoscopic procedures during their hospital stay for the management of dehydration, renal impairment, and/or electrolyte disturbances. Balloon dilatation was performed by three experienced endoscopists under deep sedation using intravenous propofol, given by an anesthesiologist. An achalasia balloon (Rigiflex TM II, Boston Scientific, Marlborough, MA, USA) was used for the dilatation of the functional gastric stenosis. The scope was advanced up to the level of the gastric stenosis under direct vision, and then by performing certain endoscopic manipulations, the scope passed through the stenosis and was advanced more distally to reach the duodenum. A metallic guidewire (Savary-Gilliard^®^ Wire Guide, Wilson-Cook Medical, Winston-Salem, NC, USA) was passed through the scope to reach the duodenum. Then, the guidewire was left in place as the scope was withdrawn. The lubricated achalasia balloon was slid over the guidewire and positioned across the gastric stenosis. It was then inflated to 30 mm for 5 min under the visualization of the reintroduced endoscopy and fluoroscopic guidance. Additional endoscopic dilatation sessions were carried out with a 35 mm and/or 40 mm balloon, with an interval of 2–4 weeks between consecutive dilatations, contingent on the clinical response of the patients. The steps of balloon dilatation for functional post-sleeve-gastrectomy stenosis are illustrated in Figure 2.

### 2.6. Definitions

Functional post-sleeve-gastrectomy stenosis was defined by the presence of a gastric twist with no evidence of the anatomical narrowing of the gastric lumen. The scope shows a deviated staple line with a clockwise rotation, and it can pass beyond the stenosis by performing certain endoscopic manipulations. Organic post-sleeve gastrectomy stenosis was considered when a real narrowing of the gastric lumen was evident. This organic stenosis cannot be traversed endoscopically, or hard friction with mucosal injury may occur when the stenosis is passed through. The term “complete clinical response” refers to the complete alleviation of the patient’s post-sleeve-gastrectomy stenosis symptoms. A partial clinical response was defined as an improvement in symptoms without a complete resolution. A non-response was confirmed if there was no improvement in symptoms following 30 mm and/or 35 mm balloon dilatation. A failure of endoscopic dilatation was defined by persistent symptoms following serial dilatations up to the maximum 40 mm balloon size. Clinical success was defined by a sustained complete clinical response at 6 months follow-up with no symptom recurrence [7,10,17,23].

### 2.7. Clinical Outcomes

The primary outcome was to evaluate the clinical response to endoscopic balloon dilatation. The secondary outcomes included the timing and presentation of gastric stenosis, hospital stay, the technical features of the dilatation procedures, and adverse events that occurred during or after dilatation sessions.

### 2.8. Statistical Analysis

A statistical analysis of patients’ data was performed using Statistical Program for Social Sciences, version 24.0. Quantitative data were expressed as mean ± standard deviation (SD). Qualitative data were expressed as frequency and percentages. 

## 3. Results

### 3.1. Patients’ Characteristics

The characteristics of the study patients are shown in Table 1. 

### 3.2. The Interval between the Primary Sleeve Gastrectomy and Diagnostic Endoscopy

The mean interval between the sleeve gastrectomy and the diagnostic EGD was 9.77 ± 5.66 with a range of 3–27 months. The endoscopic balloon dilatation was performed at the same session or within one week. Seven patients underwent radiological imaging, which was performed a few days before the endoscopy.

### 3.3. Efficacy and Safety of the Endoscopic Balloon Dilatation

A total of 45 dilatations were performed on our patients using a 30 mm balloon (22 patients, 100%), 35 mm balloon (18 patients, 81.82%), and 40 mm balloon (5 patients, 22.73%). The number of dilatation sessions required for each patient for the resolution of his/her symptoms were as follows: one dilatation session (4 patients, 18.18%), two dilatation sessions (18 patients, 81.82%), and three dilatation sessions (5 patients, 22.73%), as reported in Table 2. The balloon was left inflated for 5 min during each session. The clinical response within a two-week period, following the first balloon dilatation, was assessed as a complete clinical response (4 patients, 18.18%), partial clinical response (12 patients, 54.55%), and non-response (6 patients, 27.27%). Nineteen patients (86.36%) had achieved clinical success at six months. Failures of endoscopic dilatation (up to 40 mm balloon) were observed in three patients (13.64%). No significant adverse events were found during or following the procedures. Twenty patients (90.91%) experienced epigastric pain, and nine patients (40.91%) had a sore throat after the procedures (Table 2). 

### 3.4. Endoscopic Balloon Dilatation Sessions and Outcomes

**Figure 3:**
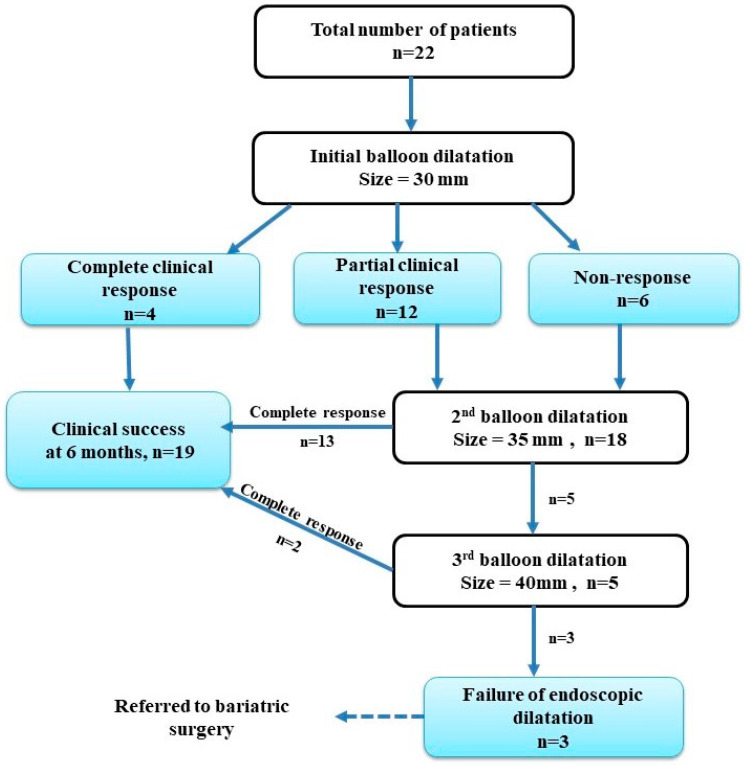
Flow chart of the endoscopic balloon dilatation sessions and outcomes in the study patients.

Details of the endoscopic balloon dilatation sessions and outcomes are outlined in Figure 3. After the first dilatation with a 30 mm balloon, 4 patients (18.18%) achieved a complete clinical response, 12 patients (54.55%) had a partial clinical response, and 6 patients (27.27%) were non-responders. Eighteen out of twenty-two patients (81.82%) who did not achieve a complete clinical response required secondary dilatation sessions with a 35 mm balloon, and thirteen of them achieved a complete clinical response. The remaining five patients did not achieve a complete clinical response and therefore underwent a third dilatation session with a 40 mm balloon. Two of them attained a complete clinical response, while three patients (13.64%) still had persistent symptoms after performing the maximum standard 40 mm balloon dilatation, and hence were considered to have not benefitted from endoscopic dilatation. They underwent a laparoscopic Roux-en-Y gastric bypass within 3 months after the last balloon dilatation, with an uneventful postoperative course and a complete alleviation of their symptoms. Clinical success at 6 months was achieved in 19 patients (86.36%). 

## 4. Discussion

Functional gastric stenosis is a potentially serious adverse event related to sleeve gastrectomy, particularly if it is not addressed early. Although its reported prevalence is low, the actual prevalence in the community is expected to be higher, due to the increasing number of sleeve gastrectomy procedures performed for obesity nowadays [24]. Many debates regarding the appropriate management of post-sleeve gastrectomy stenosis exist [19]. Being a minimally invasive procedure, endoscopic balloon dilatation has become an attractive treatment option for gastric stenosis [20,25]. The aim of the current study was to evaluate the efficacy and safety of endoscopic balloon dilatation in the management of symptomatic functional post-sleeve-gastrectomy stenosis. 

During the initial endoscopic assessment of the study patients, we observed that the majority of them had gastric stenosis at the incisura angularis, which manifested as a twist. This result was consistent with other studies demonstrating that the incisura angularis was the most common site of functional gastric stenosis, which was diagnosed as twisted stenosis with a clockwise deviation of the staple line [26,27,28,29]. 

There is still debate over many technical aspects of endoscopic balloon therapy, such as the size and type of balloon, the duration of the dilatation sessions, and their frequency. In this study, all patients were treated using achalasia balloon dilatation. As evidenced by Joo et al. and Dhorepatil et al., the achalasia balloon has a greater success rate than the controlled radial expansion (CRE) balloon because of its stiffness and wider diameter, which prevents the balloon from bending and rectifying the twisted gastric tube [30,31]. According to Shnell et al., patients treated for gastric stenosis with pneumatic dilatation using an achalasia balloon had a 100% success rate, while patients treated with CRE balloon dilatation had a 31% success rate [32]. However, as other studies have demonstrated, a CRE balloon and self-expandable metal stent were utilized in the treatment of patients with functional stenosis [10,33]. 

In this study, 30 mm was the initial balloon size utilized and inflated for 5 min/session. If a complete clinical response was not achieved after the primary session, we increased the size to 35 mm, then 40 mm, gradually and carefully in the subsequent sessions. In contrast to these findings, Alsabah et al. used the same-sized balloon for subsequent dilatations to avoid perforation [7]. Moreover, Alsabah et al. and Binda et al. performed routine repeated dilatations even with symptom improvement after the first session [7,34]. Dhorepatil et al. and Binda et al. recommended longer durations of 5–20 min for each dilatation session to achieve better clinical outcomes [31,34].

The clinical response within a two-week period following the first balloon dilatation was assessed. In total, 4 patients (18.18%) had a complete clinical response, 12 patients (54.55%) had a partial clinical response, and 6 patients (27.27%) were non-responders. Clinical success at 6 months was achieved in 19 patients (86.36%). A failure of endoscopic dilatation was observed in three patients (13.64%) who had persistent symptoms after performing the maximum standard 40 mm balloon dilatation. They had a laparoscopic Roux-en-Y gastric bypass within 3 months following the previous balloon dilatation, with an uncomplicated postoperative course and a complete remission of their symptoms.

Lorenzo et al. documented that the success rate of endoscopic treatment among their patients with functional gastric stenosis was 100% [23]. They considered both a complete and partial clinical response as an endoscopic treatment success. The variable percentages of success rates of endoscopic balloon dilatation of 95%, 56%, and 60% were reported by Abd Ellatif et al., Deslauriers et al., and Donatelli et al., respectively [10,21,29]. These wide variations among studies could be explained by the following: the difference in the type of balloons used in the studies, where achalasia balloons had higher success rates compared to CRE balloons of 100% and 31%, respectively [32]. Secondly, a long follow-up period in previous studies helped in identifying higher balloon dilatation failure rates. Thirdly, the definition of symptomatic improvement is subjective, and a partial clinical response may be regarded as a failure or a success depending on patient’s level of satisfaction [19,21].

Concerning the safety of the endoscopic balloon dilatation, we performed 45 dilatation sessions, and no significant adverse events were detected during or after the procedures. Twenty patients (90.91%) experienced epigastric pain, and nine patients (40.91%) had a sore throat following the procedures. These minor adverse events were managed conservatively, and they were resolved within 2–3 days. This was in accordance with Lorenzo et al., who reported no major complications among their patients with functional stenosis who underwent balloon dilatation [23]. On the contrary, perforation and bleeding were observed in other studies [29,35]. The high safety profile in the current study can be explained by the nature of the gastric stenosis in the study patients being functional rather than organic, as well as the gradual stepping up of dilatation size in subsequent sessions to minimize the risk of gastric perforation.

The present study has certain limitations, including its retrospective design and the small number of patients enrolled. The incidence of functional gastric stenosis in this current study could not be estimated, as the total number of sleeve gastrectomies was not available in our dataset. The non-availability of diagnostic radiological imaging in most of our patients is another limitation. The adherence to identical standardized surgical techniques could not be evaluated, as most of sleeve gastrectomies were performed in multiple diverse bariatric centers. Symptom recurrence could not be precisely evaluated due to the relatively short-term follow-up period of 6 months we adopted. The lack of objective parameters to identify the response to dilatation was considered an additional limitation; however the patients’ symptom relief was our primary end point. 

## 5. Conclusions

Based on this multi-center study, endoscopic balloon dilatation is an effective and safe minimally invasive procedure with favorable clinical outcomes in the management of symptomatic functional post-sleeve-gastrectomy stenosis. Further prospective large-scale studies, with extended follow-up periods, are required.

## Figures and Tables

**Table 1:** Characteristics of the study patients (n = 22).

Parameters	Values
Gender: females/males, n (%)	16 (72.73%)/6 (27.27%)
Age (years), (mean ± SD)	33.86 ± 8.82
BMI (kg/m^2^), (mean ± SD)	31.76 ± 2.86
Smoking, n (%)	4 (18.18%)
Comorbidities	
Diabetes mellitus, n (%)	2 (9.09%)
Hypertension, n (%)	1 (4.55%)
Knee osteoarthritis, n (%)	1 (4.55%)
Presenting symptoms	
Vomiting, n (%)	22 (100%)
Post-prandial abdominal pain, n (%)	14 (63.64%)
GERD symptoms, n (%)	7 (31.82%)
Admitted patients prior to dilatation, n (%)	11 (50%)
Duration of hospital stay (days), (mean ± SD)	3.91 ± 0.83
Radiological imaging, n (%)	7 (31.82%)
Interval between sleeve gastrectomy and endoscopy (months) (mean ± SD)	9.77 ± 5.66
Diagnostic endoscopic findings	
Hiatus hernia, n (%)	6 (27.27%)
Reflux esophagitis, n (%)	4 (18.18%)
Staple line deviation, n (%)	22 (100%)
Stenosis at incisura angularis, n (%)	20 (90.91%)

SD, standard deviation; BMI, body mass index; GERD, gastroesophageal reflux disease.

**Table 2:** Efficacy and safety of the endoscopic balloon dilatation in functional post-sleeve-gastrectomy stenosis.

Parameters	n = 22	%
Balloon size		
30 mm	22	100%
35 mm	18	81.82%
40 mm	5	22.73%
Number of dilatation sessions/patient		
One dilatation session	4	18.18%
Two dilatation sessions	18	81.82%
Three dilatation sessions	5	22.73%
Clinical response after initial dilatation (at 2 weeks)		
Complete clinical response	4	18.18%
Partial clinical response	12	54.55%
Non-response	6	27.27%
Clinical success (at 6 months)	19	86.36%
Failure of endoscopic dilatation	3	13.64%
Adverse event after dilatation		
Bleeding	0	0%
Perforation	0	0%
Epigastric pain	20	90.91%
Sore throat	9	40.91%

## Data Availability

The data sets used in the present study are available from the corresponding author upon reasonable request.

## Abbreviations

EGD	Esophagogastroduodenoscopy
BMI	Body mass index
SD	Standard deviation
GERD	Gastroesophageal reflux disease
CRE	Controlled radial expansion
